# Supplementary material for: Interactions between time on diet, antibiotic treatment, and liver abscess development on the fecal microbiome of beef cattle
Source: Anim Microbiome. 2025 May 12;7:45. doi: 10.1186/s42523-025-00413-z (PMC12067690; doi:10.1186/s42523-025-00413-z)
Supplement: Supplementary file 2 — Supplementary Material 2. [file 42523_2025_413_MOESM2_ESM.pdf]

Supplementary tables

Supplementary table 1. Diet formulations and nutrient analysis for study 1.

**Diet ingredients**					
Ingredient Name	Units	**Starter**	**Home Calf**	**Step Up**	**Finish**
Alfalfa Hay	%	28.5	19	11	3
Corn Ground	%	4.4	4.4	4.4	4.4
Corn Rolled	%	7.6	2.6	2.3	2.6
Corn Syrup	%	22	22	21	15
Distillers	%			2.3	3
Earlage 75% Dry	%	29	28	32.5	39
Non-Med FL-721	%	4.5	4.5	4.5	4.5
Tallow	%			1	2
Wheat	%		13.5	17	25.5
Wheat Straw	%	4	6	4	1
**Nutrient analysis**					
Item	Units	**Starter**	**Home Calf**	**Step Up**	**Finish**
Ration Dry Matter	%	69.63	99.6	70.12	72.53
Nem	Mcal/cwt	76.61	79.52	87.9	97.61
Neg	Mcal/cwt	49.31	51.89	60.11	69.45
Fat	%	3.53	3.38	5.05	6.28
ADF	%	19.78	18.72	15.65	11.48
NDF	%	30.12	28.58	25.8	21.52
Crude Protein	%	14.2	13.94	13.95	13.34
NPN (DM)	%	3.02	3	3	2.9
RUP (CP) (book value)	%	24.17	23.92	28.02	30.23
RDP (CP) (book value)	%	75.83	76.08	71.98	69.77
Calcium	%	1.11	0.97	0.85	0.69
Phosphorus	%	0.39	0.4	0.43	0.42
Calcium Phosphorus Ratio		2.88	2.42	1.97	1.63
Magnesium	%	0.26	0.25	0.25	0.22
Potassium	%	1.51	1.31	1.08	0.75
Sulfur	%	0.22	0.21	0.21	0.19
Salt	%	0.5	0.48	0.47	0.45
Sodium	%	0.25	0.25	0.26	0.24
Zinc ppm	ppm	78.41	82.89	85.86	84.58
Manganese	ppm	44.39	48.17	50.18	50.09
Copper	ppm	24.77	24.86	26.89	24.99
Cobalt	ppm	0.47	0.42	0.4	0.38
Iodine	ppm	0.59	0.58	0.58	0.55
Selenium	ppm	0.39	0.4	0.39	0.38
Vitamin A	IU/lb	2,640.30	2,627.65	2,621.88	2,534.55
Vitamin D	IU/lb	293.37	291.96	291.32	281.62
Vitamin E	IU/lb	3.26	3.24	3.24	3.13
Forage Dry Matter	%	44.71	35.39	23.74	10.82
Concentrate Dry Matter	%	55.29	64.61	76.26	89.18

Supplementary table 2. Diet formulations and nutrient analysis for study 2.

Diet ingredients					
Ingredient Name	Units	Starter Ration	Home Calf	Step Up	Finish
Alfalfa Hay	%	27.5	27.7	16.5	2.8
Corn Rolled	%	12	10.2	13.5	20
Corn Syrup	%	22.4	21	19	14
Distillers	%				2
Earlage	%	27	18	28.5	33.5
Non-Med FL-721	%	2	4.1	4.1	5
Tallow	%			1	1.9
Water	%	6	6	5	5
Wheat	%		13	11.8	13
Wheat Straw	%	3.5			2.8
**Nutrient analysis**					
Item		Starter Ration	Home Calf	Step Up	Finish
Ration Dry Matter	%	64.39	65.64	65.4	67.24
Nem	Mcal/cwt	76.12	80.22	89.24	96.1
Neg	Mcal/cwt	50.11	52.3	61.14	67.94
Fat	%	3.57	3.34	5.05	6.54
ADF	%	18.56	16.58	13.37	10.86
NDF	%	30.62	28	23.18	21.12
Crude Protein	%	13.13	15.29	14.03	13.37
NPN (DM)	%	1.45	2.92	2.93	3.47
RUP (CP) (book value)	%	24.05	19.83	21.85	27.23
RDP (CP) (book value)	%	75.95	80.17	78.15	72.77
Calcium	%	0.83	1.09	0.91	0.79
Phosphorus	%	0.41	0.43	0.41	0.4
Calcium Phosphorus Ratio		2.03	2.54	2.22	2
Magnesium	%	0.25	0.26	0.23	0.21
Potassium	%	1.51	1.41	1.08	0.76
Sulfur	%	0.2	0.21	0.19	0.16
Salt	%	0.29	0.51	0.5	0.57
Sodium	%	0.14	0.24	0.23	0.25
Zinc ppm	ppm	51.34	82.34	81.81	91.64
Manganese	ppm	31.12	44.91	41.47	43.1
Copper	ppm	18.32	23.37	22.45	24.43
Cobalt	ppm	0.41	0.49	0.49	0.51
Iodine	ppm	0.32	0.59	0.57	0.63
Selenium	ppm	0.29	0.43	0.36	0.37
Vitamin A	IU/lb	1,629.01	2,551.65	2,561.22	3,037.88
Vitamin D	IU/lb	141	283.52	284.58	337.64
Vitamin E	IU/lb	1.57	3.15	3.16	3.75
Forage Dry Matter	%	45.85	36.97	28.51	12.67
Concentrate Dry Matter	%	54.15	61.03	73.49	87.33

Supplementary figures

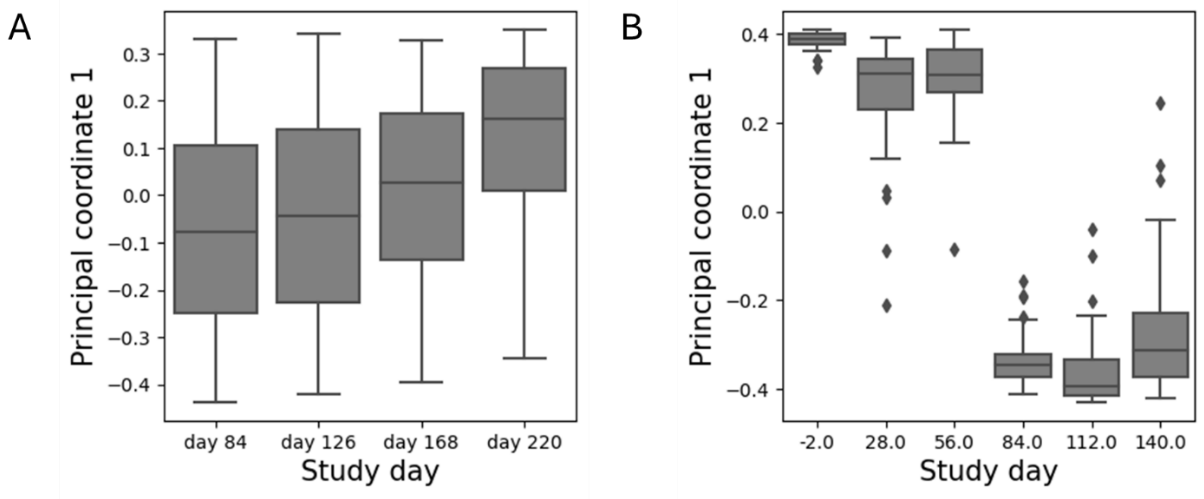

Supplementary figure 1. Relationship between the time of fecal sample collection and corresponding locations along the first coordinate of the principal coordinates analysis (PCoA) of Bray-Curties dissimilarities between samples in study 1 (A) and study 2 (B). Boxes represent the interquartile range, and whiskers extend to minimum and maximum values, points outside 1.5 times the interquartile range are plotted individually.

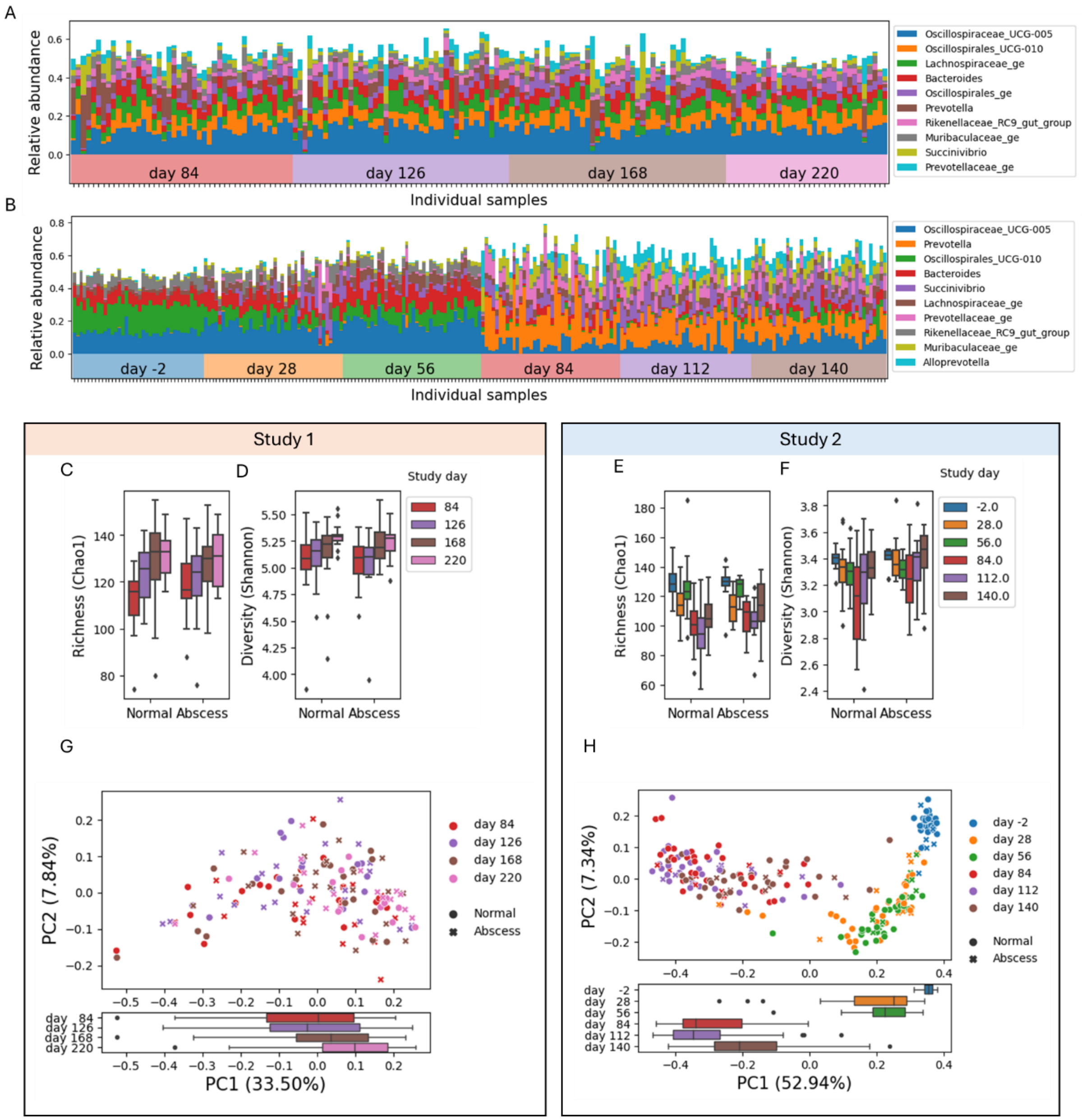

Supplementary figure 2. Composition of the fecal microbiome of steers in study 1 and study 2. A. Relative abundances of major genera of bacteria detected in samples passing quality thresholds in study 1. Each bar represents an individual sample. Samples are grouped according to the time of sample collection. **B.** Like A but for study 2. **C, D**. the Chao1 and Shannon alpha diversity indexes for genera in samples from normal or abscessed steers at different study days from study 1. Boxes represent the interquartile range, and whiskers extend to minimum and maximum values, points outside 1.5 times the interquartile range are plotted individually. **E, F.** Like C, D but for study 2. **G.** The first two principal coordinates calculated on the Bray-Curtis dissimilarity of the relative abundances of genera in samples from study 1. Colors represent different fecal sample collection times and symbols indicate abscess diagnosis of corresponding animals. Numbers in parentheses indicate the variance explained by the corresponding principal coordinate. The boxplot shows the distribution of samples collected at different time points on the first principal coordinate. **H.** Like G but for study 2.

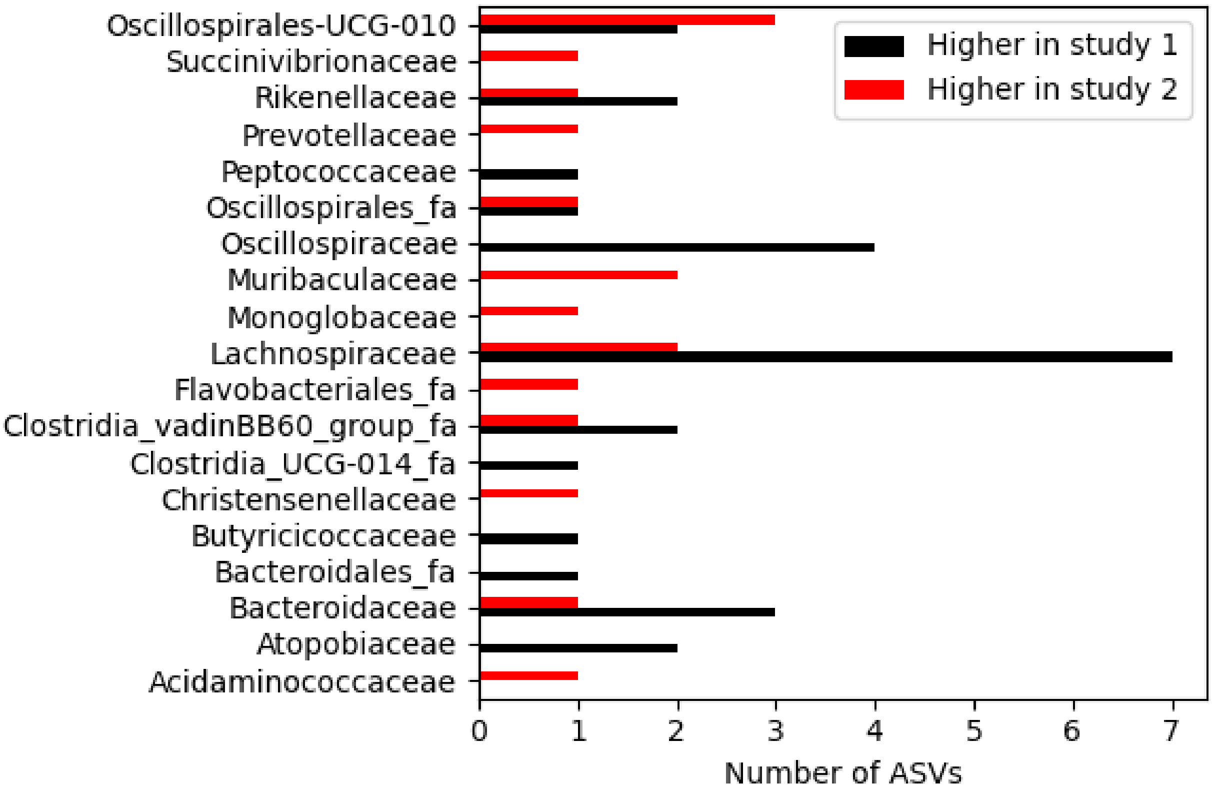

Supplementary figure 3. Differentially abundant ASVs between studies. The number of ASVs in each taxonomic family significantly more abundant in one study compared to the other. The comparison was performed between control samples at the latest time points of each study (day 220 for study 1 and day 140 for study 2). Significant ASVs were calculated with metamicrobiomeR using the GAMLSS model with study as the main comparison variable and adjusting for liver abscess diagnosis.
